# Rapid, Simple, and Highly Specific Detection of *Streptococcus pneumoniae* With Visualized Recombinase Polymerase Amplification

**DOI:** 10.3389/fcimb.2022.878881

**Published:** 2022-06-02

**Authors:** Fang Wang, Yan Wang, Xia Liu, Lei Wang, Kun Wang, Chenglai Xu, Guanhong Huang, Xuzhu Gao

**Affiliations:** Department of Central Laboratory, The Second People’s Hospital of Lianyungang City (Cancer Hospital of Lianyungang), Affiliated to Jiangsu University, Lianyungang, China

**Keywords:** recombinase polymerase amplification, rapid assay, false-positive signal, *Streptococcus pneumoniae*, lateral flow strip

## Abstract

*Streptococcus pneumoniae* is a major pathogen that causes microbiological illness in humans. The introduction of polyvalent vaccines has resulted in a significant decrease in pneumococcal-related mortality. However, pneumococcal infections continue to be a leading cause of death in children under the age of 5 and adults over the age of 65 worldwide. A speedy and highly sensitive diagnostic tool is necessary for routine adoption to adequately manage patients and control the spread of infection. In this study, we investigated a new nucleic acid amplification technique, isothermal recombinase polymerase amplification (RPA), which amplifies DNA at 37°C under isothermal conditions with high specificity, efficiency, and rapidity. Using the autolysin gene *lytA* as the molecular diagnostic target, an RPA primer-probe combination was designed and optimized for the detection of *S. pneumoniae*. This RPA reaction produced amplification products labeled with specific chemical markers, to be detected with gold-nanoparticle-based lateral flow strips (LFS), reducing the reliance on equipment and trained personnel. The high specificity of the RPA-LFS technique was demonstrated with the specific detection of 22 strains of *S. pneumoniae* but not 25 closely related pathogenic bacteria. The assay showed good sensitivity, and detected *S. pneumoniae* down to 3.32 colony-forming units/μL. When used on clinical samples, the assay provided accurate and consistent results compared with PCR. The compliance with the culture-biochemistry method was 98.18% and the kappa index was 0.977. These results reveal that the RPA–LFS test significantly improved *S. pneumoniae* identification, particularly in resource-limited areas.

## Introduction

*Streptococcus pneumoniae* is a Gram-positive, non-flagellated bacterium, often arranged in pairs or short chains of cells (Ye et al., 2018; Paton and Trappetti, 2019). It is widely distributed in nature and often colonizes the mucous membranes of the human upper respiratory organs, mainly targeting immunocompromised people, such as children and the elderly. This bacterium causes pneumonia, meningitis, otitis media, and other invasive diseases after infection, and the annual global morbidity and mortality rates of *S. pneumoniae* infections are very high (Kadioglu et al., 2008; Reynolds et al., 2010; Yu et al., 2019; Zhao et al., 2020). This bacterium is the most common pathogen causing community-acquired pneumonia in clinical practice, and fast and correct pathogenic identification is critical in the selection of clinical therapeutic medications and the construction of treatment strategies (Thummeepak et al., 2015; Arushothy et al., 2020).

The early detection of a clinical infection with a timely and accurate diagnosis in the early stages of the patient’s illness allows the appropriate treatment to be administered. However, the current gold standard methods for detecting *S. pneumoniae* are phenotype based, and include culture-based, microscopy-based, and biochemical identification methods (Suárez and Texeira, 2019). Because *S. pneumoniae* growth and identification typically take more than 2 days, positive identification may occur late in the course of infection, and a delayed diagnosis may result in a bad prognosis for individuals infected with this pathogen (Petti et al., 2005). As a result, it is critical to develop and verify a speedy and precise approach to identifying *S. pneumoniae*. Several non-culture methods for detecting *S. pneumoniae* have been developed, including mass spectrometry, immunoassay, PCR, and real-time PCR (El Aila et al., 2010; Park et al., 2010; Lang et al., 2015; Iroh Tam et al., 2018; Kim et al., 2019; Kann et al., 2020). These tests can save considerable time compared with the gold standard culture methods. However, such analyses require skilled technicians and/or sophisticated equipment, which may be unavailable in some situations.

**Figure 1:**
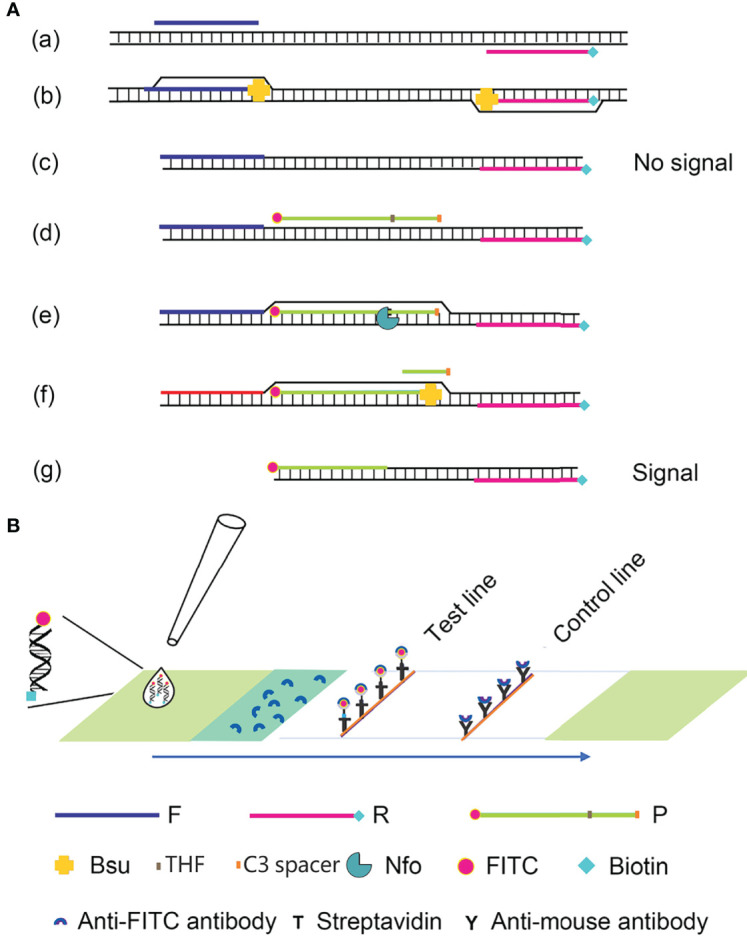
Schematic diagram of the RPA-LFS method. **(A)** The RPA amplification principle. Base pairing is represented as short vertical lines between DNA strands, and DNA strands are represented as horizontal lines. Base pairing is represented by a short vertical line connecting two DNA strands. **(B)** A schematic representation of the lateral flow strip (LFS) operation. The shapes and the molecules that represent them are listed below the graphic.

Recombinase polymerase amplification (RPA) is a recombinase-polymerase-mediated amplification technique that mimics DNA replication in living organisms and allows the isothermal amplification of target DNA fragments at room temperature (Piepenburg et al., 2006). The technique relies on three enzymes: the T4-phage-encoded recombinase proteins uvsX and uvsY, the single-stranded binding protein gp32, and the *Bacillus subtilis* (*Bsu*) DNA polymerase. The recombinase proteins bind to the primers to form DNA nucleoprotein microfilaments, which bind to complementary DNA fragments, which then hybridize tightly. With the help of the single-stranded binding protein, the strands of the template DNA begin to separate, and are extended by the *Bsu* DNA polymerase, which exponentially amplifies the target region on the template. The entire process can be completed in 20–30 min at 37–42°C (Wang et al., 2017; Dong et al., 2020). Compared with PCR, the process does not require high temperature denaturation or low temperature annealing, making the reaction simple, fast, and efficient. The labeled amplification products are detected visually by combining RPA with a lateral flow strip (LFS) of encapsulated gold nanoparticles (AuNPs), and the color signal can be observed semiquantitatively on the LFS with the naked eye (Wang et al., 2019). This technique simplifies the detection process and allows the *in situ* detection of the result without instruments. RPA–LFS has been successfully utilized to identify methicillin-resistant *Staphylococcus aureus*, *Mycobacterium tuberculosis*, *Candida albicans*, *Klebsiella pneumoniae*, and other pathogenic microorganisms (**Figure 1**) (Hu et al., 2020; Wang et al., 2020; Wang et al., 2021a; Wang et al., 2021b).

In this study, a rapid and sensitive field assay for *S. pneumoniae* was developed using RPA combined with the LFS technology. The method was based on primers and a probe designed to complement the *S. pneumoniae* autolysin gene (*lytA*) and the experiment was completed in 30 min at 37°C (Kersting et al., 2018). The specificity of the method was verified by testing it against 22 clinical isolates of *S. pneumoniae* and 25 other common pathogenic strains. The sensitivity of the RPA-LFS technique was tested in 10 independent trials, and the limit of detection (LOD) was 3.32 colony-forming units (CFU)/reaction. Finally, the established RPA-LFS assay for *S. pneumoniae* was used to analyze clinical specimens, with accurate results that were consistent with those achieved with PCR. In conclusion, we developed a rapid, specific, and sensitive assay for the detection of *S. pneumoniae* with RPA-LFS, with potential applications in the preliminary medical diagnosis of *S. pneumoniae* in remote and resource-limited areas.

## Materials and Methods

### Standard Strains and Clinical Isolates

A standard strain of *S. pneumoniae* (American Type Culture Collection ATCC 49619) was used to establish the RPA-LFS method for detecting *S. pneumoniae*. Twenty-two clinical isolates of *S. pneumoniae* were obtained from sputum samples from the lower respiratory tract, with serotypes 19F, 19A, 14, 23F, and 6A, respectively. To validate the specificity of the RPA-LFS approach, isolates of 25 other common pathogens (including *Escherichia coli*, *Haemophilus influenzae*, *Acinetobacter baumannii*, *Pseudomonas aeruginosa*, *Enterococcus Faecium*, *Staphylococcus aureus*, *Klebsiella pneumoniae*, *Enterococcus faecalis*, *Serratia marcescens*, *Burkholderia cepacia*, *Candida albicans*, *Candida krusei*, *Vibrio Parahemolyticus*, *Streptococcus lactis*, *Bacillus cereus*, *Salmonella enterica*, *Morganella fulton*, *Coagulase negative Staphylococci*, *Bacillus mirabilis*, *Stenotrophomonas maltophilia*, *Streptcoccus pyogenes*, *Streptcoccus agalactiae*, *Streptcoccus dysgalacitae*, *Streptcoccus mitis*, and *Streptcoccus oralis*) were employed. At the Department of Laboratory of the Second People’s Hospital of Lianyungang City, all strains were identified using the reference culture-biochemical approach.

One hundred and ten clinical specimens were collected from patients, including 80 respiratory sputum and 30 invasive specimens (16 blood, 10 cerebrospinal fluid, and 4 peritoneal fluid), which were provided by Lianyungang Second People’s Hospital. The positive strains isolated were serotyped by the capsular swelling test and were 19F, 19A, 14, 23F, 9V, 6B, 6A.

### Extraction of Bacterial Genomes

Genomic DNA was obtained using the Bacterial Genomic DNA Extraction Kit (Tiangen Biochemical Technology Co., Ltd, China) and stored at -20°C as a backup.

### Primer Design for RPA Reactions

Specific RPA primers based on the species-specific *S. pneumoniae* autolysin gene (*lytA*) sequence was designed with the Primer-BLAST online design software from the National Center for Biotechnology Information (NCBI). The primer design parameters were: primer size 30–35 bp, product size 100–500 bp, GC content 20%–80%, Tm 50–100°C, and organism *S. pneumoniae*. All other parameters were set to the default values. Five primer pairs were selected (General Biologicals Ltd, Anhui, China) for testing.

### RPA Procedure

To screen the best forward and reverse primer pairs, RPA amplification was performed using the TwistAmp Liquid DNA amplification Kit (TwistDx Inc., Maidenhead, United Kingdom). Each 50 μL mixture contained 25 μL of 2× reaction buffer, 5 μL of 10× basic mix, 2.5 μL of 20× core mix, 2.1 μL of forward primers (10 μM), 2.1 μL of reverse primers (10 μM), 9.8 μL of ddH_2_O, and 1 μL of the template genome. To ensure that all reaction systems reacted at the same time, 2.5 μL of 280 mM magnesium acetate was added to the PCR tube caps and transiently centrifuged into all reaction tubes. The reaction system was vortexed and immediately incubated at 37°C in a thermostat heater for 30 min. The amplification products were purified using the DNA Purification Kit (Tiangen Biochemical Technology Co., Ltd, Beijing, China) and detected using 1.5 percent agarose gel electrophoresis.

### RPA-LFS Probe Design

RPA amplification requires the same pair of forward and reverse primers as PCR amplification. When LFS is used as the endpoint visual readout for an amplified DNA target, a probe must be designed downstream from the forward primer. The 5′ end of the probe was labeled with fluorescein isothiocyanate (FITC), and a tetrahydrofuran (THF) site was included in the middle of the probe which is then closed at the end. When a certain amount of product accumulated in the reaction system, the probe bound to the product, at which point the nfo enzyme in the reaction system recognized the THF site and cleaved it. Because the *Bsu* polymerase had strand replacement activity, it displaced the DNA strand after the THF site and began amplification. The final product obtained had FITC on one end and biotin on the other (Wang et al., 2018).

We used the Primer Premier 5 software to design specific probes complementary to sites between the sequences targeted by the forward and reverse primers. Theoretically, the formation of a dimeric structure between the probe and the reverse primer should be avoided. The parameters required to do so are: (1) a probe of 46–51 bp, Tm of 57–80°C, and GC content of 20%–80%; (2) The maximum primer dimer fraction is set to nine, the maximum hairpin fraction is set to nine, the maximum poly-X is set to five, and all other parameters are set to their default values. (3) The 5′ end of the probe was tagged with FITC, the 3′ end was blocked with the C3 spacer, and the middle base of the probe was replaced with THF, with at least 30 nucleotides before the THF site and at least 15 nucleotides after it; (4) the reverse primer’s 5′ end was labeled with biotin.

### RPA-LFS Procedure

To determine the optimal probe and primer combinations, the RPA-LFS assay was done using the TwistAmp DNA Amplification Nfo Kit (TwistDx Inc.). Each 50 μL reaction system included 2.1 μL of RPA forward and reverse primers (10 μM), 0.6 μL of RPA probe (10 μM), 11.2 μL of ddH_2_O, 29.5 μL of hydration buffer, 2 μL of genomic DNA, and dried enzyme pellets. 2.5 μL of 280 mM magnesium acetate was added to the tube caps to guarantee that all of the reaction systems started at the same time. The tubes were briefly centrifuged before being incubated for 30 min in a constant-temperature heater set to 37°C. Then, within 5 min, 5 μL of the amplified product was visually evaluated with LFS (Ustar BioTechnologies Ltd, Hangzhou, China). On the LFS, two red lines were displayed: the control line (top) and the test line (bottom). The control line was present in all tests to guarantee the LFS’s validity, whereas the test line was only shown in positive reactions.

### Specificity Assay

RPA-LFS specificity for *S. pneumoniae* was tested using genomic DNA from 22 clinical isolates of *S. pneumoniae* and 25 common pathogenic bacteria.

### Limit of Detection (LOD) Assay

A 10-fold dilution series of the *S. pneumoniae* genome, corresponding to numbers of bacteria ranging from 3 × 10^4^ CFU to 3 × 10^−1^ CFU was prepared for the RPA-LFS reaction. The LOD of the method was determined with a probit regression analysis of 10 independent experiments.

### Polymerase Chain Reaction

PCR primers were designed based on the *S. pneumoniae* autolysin *lytA* gene, and the primer sequences are in **Table 1**. 25 μL of the reaction system was used, including 12.5 μL of PCR Mix (Tiangen Biochemical Technology Co., Ltd., Beijing, China), 0.5 μL (10 µM) each of forward and reverse primers, 1 μL of template, and 10.5 μL of ddH_2_O. The cycling procedure was 95°C pre-denaturation for 5 min, followed by 30 cycles including denaturation at 95°C for 30 s, binding at 55°C for 30 s, extension at 72°C for 1 min, and finally extension at 72°C for 5 min. amplification of the products was detected by 1.5% agarose gel electrophoresis.

**Table 1:** Primers and probes tested in this study.

Name	Sequence (5’-3’)	Length (bp)	Amplicon size (bp)
lytA-1-F	ACAGAATGAAGCGGATTATCACTGGCGGAAAGA	33	351
lytA-1-R	GGATAAGGGTCAACGTGGTCTGAGTGGTTGTTTG	34	
lytA-2-F	CCGTACAGAATGAAGCGGATTATCACTGGCG	31	355
lytA-2-R	GGATAAGGGTCAACGTGGTCTGAGTGGTTGTTTG	34	
lytA-3-F	CAGAATGAAGCGGATTATCACTGGCGGAAAG	31	369
lytA-3-R	CCATTTAGCAAGATATGGATAAGGGTCAACG	31	
lytA-4-F	CATTGTTGGGAACGGTTGCATCATGCAGGTA	31	281
lytA-4-R	CGTGGTCTGAGTGGTTGTTTGGTTGGTTATTCG	33	
lytA-5-F	GCAGGTTTGCCGAAAACGCTTGATACAGGG	30	154
lytA-5-R	CATGCTTAAACTGCTCACGGCTAATGCCCCAT	32	
P1	FITC-AATCTAGCAGATGAAGCAGGTTTGCCGAAA[THF] CGCTTGATACAGGGA-/C3-spacer/	46	125
P2	FITC-CAATCTAGCAGATGAAGCAGGTTTGCCGAA[THF] ACGCTTGATACAGGG-/C3-spacer/	46	113
lytA-2-mR	Biotin-GGATAAGGGTCAACGTGGTCTGAGTGGTTGGTTG	34	/
lytA-4-mR	Biotin-CGTGGTCTGAGTGGTTGTTTGGTTGGTTAGTCG	33	/
mP1	FITC-AGTCTAGCAGATGAAGCAGGTTTGCCGAAA[THF] CGCTAGATACAGGGA -/C3-spacer/	46	/
mP2	FITC-CAATGTAGCTGATGAAGCAGGTTTGCTGAA[THF] ACGCTTGATACAGGG-/C3-spacer/	46	/
PCR-lytA-F	CAGATTTGCCTCAAGTCGGCGTGC	24	691
PCR-lytA-R	CCTGTAGCCATTTCGCCTGAGTTGTC	26	

Sequences modified with base substitutions. Modified bases are in red. F and R represent forward and reverse primers, respectively.

### Examination of Clinical Specimens

The RPA-LFS method was evaluated on clinical specimens to determine its compliance with both traditional culture–biochemical methods and PCR. Clinical specimens were cultured at 37°C for 18–48 hours on selective media, including blood culture bottles and columbia blood plate. Bacterial identification was carried out using the VITEK^®^ 2 system (bioMérieux, Marcy-l’Étoile, France), with further biochemical assays carried out if necessary. For PCR, the primers were designed to amplify the *lytA* gene. The compliance rate between the different methods was calculated with the formula: ([number of positive samples detected with both methods + number of negative samples detected with both methods]/total number of samples) × 100%. The kappa index was calculated to evaluate this test.

## Results

### Design and Screening of Primer Sets for the RPA System

**Figure 2:**
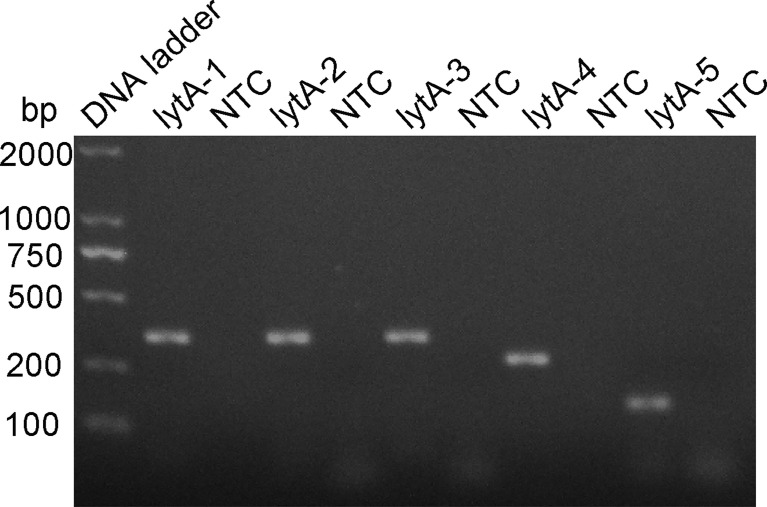
Screening RPA primer sets. The primer pairs lytA-1 to lytA-5 were screened with the RPA method using genomic DNA from standard *S. pneumoniae* strains as the templates. An NTC for each primer set was included as the negative control and 1.5% agarose gel electrophoresis was used to analyze equal volumes (5 μL) of the amplified products.

The rational design of primers for detecting *S. pneumoniae* started with a BLAST search with the *lytA* gene sequence. The primers were designed to match the *S. pneumoniae* sequence only. As shown in **Table 1**, five pairs of primers, lytA-1, lytA-2, lytA-3, lytA-4, and lytA-5, were designed to hybridize with the *lytA* gene. The basic RPA reaction was carried out using the genomic DNA of standard *S. pneumoniae* strains as a template, and the products were identified using agarose gel electrophoresis. All five primer sets (lytA-1 to lytA-5) produced distinct target bands with diameters of 456, 456, 456, 275, and 204 bp, respectively, and although there were no nonspecific amplification bands in the NTC, primer dimers of 100 bp were still present (**Figure 2**). However, primer pairs lytA-2 and lytA-4 amplified brighter target bands with fewer primer dimers. Therefore, we selected primer pairs lytA-2 and lytA-4 to design the probes for the RPA–LFS systems.

### Modification and Determination of Optimal Primer–Probe Combinations for RPA-LFS

**Figure 3:**
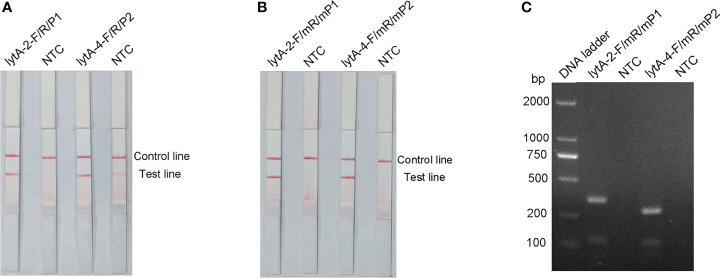
Performance of the primer-probe sets tested with the RPA-LFS system. **(A)** Showing the LFS assay results of RPA amplification products before mismatch. **(B)** Showing the LFS assay results of RPA amplification products after mismatch. **(C)** Agarose gel results. The name of each primer-probe set is shown above the corresponding strip. NTC strip is the no-template control for the corresponding RPA. The positions of the test and control lines are marked on the right side of the bars. Reactions were performed at 37°C for 30 min. This image represents the results of three independent experiments.

P1 and P2 probes were designed to bind within the sequences amplified by the lytA-2 and lytA-4 primer pairs, respectively, and RPA-LFS tests were performed to determine the amplification performance and false-positive results of the primer–probe combinations lytA-2/F/R/P1 and lytA-4/F/R/P2. **Figure 3A** depicts the results. Both primer-probe combinations produced the expected positive results (visible red bands on both the test and control lines), suggesting that both combinations amplified the target sequence effectively. However, they both also generated a weak red band on the test line in the NTC, indicating false-positive signals for both primer-probe combinations.

The FITC- and biotin-labeled RPA products produced by the probe and reverse primer are particularly recognized by the LFS. Therefore, the RPA-LFS probe should be designed in such a way that the NTC signal is entirely suppressed. Previous research has demonstrated that the RPA reaction can tolerate minor mismatches between primers or probes and the template (Daher et al., 2015). The Primer Premier 5 software was used to examine the potential for probe-reverse primer dimers, and mismatches were inserted at sites with more than five continuous bases or more than three bases at the 3′ end. **Table 1** shows the sequences of the modified reverse primer (mR) and probe (mP), with the replaced bases highlighted in red. The modified probes and primers were then used in the RPA-LFS assay. When the *lytA* gene was amplified from *S. pneumoniae* genomic DNA, both primer-probe pairs showed no signal on the detection line in the NTC group and a significant signal on the detection line in the group containing *S. pneumoniae* genomic DNA (**Figure 3B**). Because the number of mismatched bases in the lyt-2-F/mR/mP1 combination was small, we assumed that this combination performed better. Analysis of the RPA amplification products with agarose gel electrophoresis revealed two clear bands for both primer–probe combinations, representing the amplification products generated with the forward and reverse primers and with the probe and the reverse primer (**Figure 3C**). Overall, the best primer-probe combination for RPA–LFS detection of *S. pneumoniae* was lyt-2-F/mR/mP1.

### Specificity Analysis of the RPA-LFS Assay

**Figure 4:**
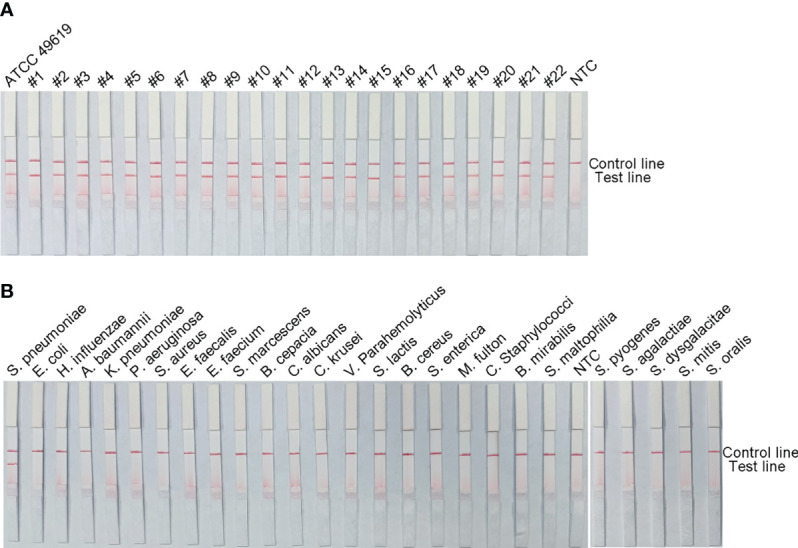
The RPA-LFS assay’s specificity. *S. pneumoniae* clinical isolates **(A)** and other common pathogens **(B)** were tested. The positive control was *S. pneumoniae* (ATCC 49619). Each bacterium’s species name is shown at the top of each strip. The NTC strip is a no-template control. The reactions were carried out for 30 minutes at 37°C.

To verify the inclusiveness and specificity of the primer–probe combination lyt-2-F/mR/mP1, RPA-LFS was used to analyze 22 clinical isolates of *S. pneumoniae* and 25 other pathogenic bacteria. **Figure 4** shows that when isolated *S. pneumoniae* genomic DNA was used as the template, a clear positive signal appeared on the test line, however no bands showed on the test line when genomic DNA from any other common respiratory infection was used as the template. These results indicated that the RPA-LFS assay system established here was highly specific for *S. pneumoniae* and does not cross-react with other pathogens.

### LOD of the RPA-LFS Assay

**Figure 5:**
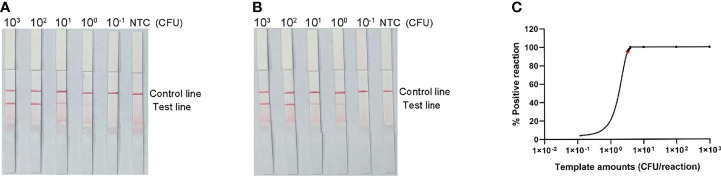
Determination of the limit of detection (LOD) of the *S. pneumoniae* RPA-LFS assay. **(A)** The LOD of the established *S. pneumoniae* RPA-LFS assay was determined from 10 independent assays using the serially diluted genomic DNA of *S. pneumonia*, equivalent to 10^4^ to 10^−1^ CFU. Images show the results of the RPA-LFS assays, and the amount of template is indicated at the top of the bar graph. **(B)** The group with 10 ng human genomic DNA added in addition to the P. gingivalis genomic DNA. **(C)** Probit regression analysis was performed on data collected from 10 replicates, using the SPSS software.

The detection limit of the RPA-LFS assay was assessed using a 10-fold dilution of inactivated *S. pneumoniae* culture as the template, comparable to bacterial counts ranging from 3 × 10^4^ CFU to 3 × 10^−1^ CFU (1 μL, reaction volume of 50 μL). A clear red band was visible on the detection line at 3 × 10^4^ CFU, and the signal diminished as the amount of template decreased, disappearing altogether in the 3 × 10^−1^ CFU sample (**Figure 5A**). To test whether the system was resistant to interference from human genomes, 10 ng of human DNA were added to the RPA reaction along with dilutions of *S. pneumoniae* genomic DNA. The detection sensitivity was not affected by human DNA (**Figure 5B**). Not all assays produced positive results when template equivalent to 3 × 10^0^ CFU (nine positive results in 10 samples) or 3 × 10^−1^ CFU (one positive results in 10 samples) were used. To confirm the LOD of the RPA–LFS assay more accurately, a probit regression analysis was performed on data from 10 independent assays. The statistical analysis was performed with the SPSS software. The LOD for each reaction was 3.32 CFU, with 95% probability (**Figure 5C**).

### Application of *S. pneumoniae* RPA-LFS to the Identification of Clinical Specimens

To assess the clinical utility of the established RPA-LFS detection system, 110 clinical specimens were collected from patients and examined with the RPA-LFS method, a PCR method, and a culture-biochemical approach at the Second People’s Hospital of Lianyungang. As indicated in **Table 2**, 31 of 110 samples tested positive for *S. pneumoniae* using the RPA-LFS and PCR methods, whereas 30 of 110 tested positive using the culture-biochemistry method. The established RPA-LFS assay was 100 percent compliant with the PCR technique. The RPA-LFS approach had a 98.18% compliance rate with the conventional culture-biochemical method, and the estimated kappa index was 0.977, suggesting that the difference between the two methods was not statistically significant (p > 0.05). These results demonstrated the feasibility and reliability of the highly specific and sensitive *S. pneumoniae* RPA-LFS when applied to clinical samples from patients.

**Table 2:** Assay performance of the RPA-LFS system, PCR and culture-biochemical method.

Method	positive	negative	Total	Amplification time
RPA-LFS	31	79	110	35 min
qPCR	31	79	110	90 min
Culture-biochemical method	30	80	110	2 d

## Discussion

*Streptococcus pneumoniae* can be found in the nasopharynx of healthy adults as well as children, and has a wide clinical distribution. It is usually cultured in a medium of blood or serum, where it forms round, grayish-white colonies (Li and Zhang, 2019). It can be spread in airborne droplets and is distributed in greater amounts in places where interpersonal contact rates are high, such as hospitals and military barracks. Infection rates are higher in the elderly, children, and those with low resistance. Consequently, *S. pneumoniae* is the main pathogen causing severe pediatric pneumonia. The early detection and diagnosis of pathogenic clinical infections with timely, effective, and accurate testing in the early stages of a patient’s illness allow the correct treatment to be administered (Allan et al., 2016). However, a diagnosis is traditionally made by culturing the bacterium, which is not only time-consuming, but is also susceptible to contamination with other bacteria during the culture process, compromising the accuracy of detection and therefore the diagnosis. In consequence, the choice of treatment plan and the recovery of the patient will be seriously affected. Therefore, a reliable diagnostic method that can rapidly, sensitively, and specifically identify *S. pneumoniae* in a near-patient setting could play an important role in reducing the morbidity and mortality associated with pneumococcal disease, especially in developing countries.

Because they do not require temperature cycling, *in vitro* isothermal nucleic acid amplification techniques are gaining popularity in molecular diagnostics. Transcription-mediated amplification (TMA), nucleic-acid-sequence-based amplification (NASBA), helicase-dependent amplification (HDA), rolling loop amplification (RCA), loop-mediated isothermal amplification (LAMP), and chain displacement amplification (SDA) are the most common isothermal amplification techniques used today (Walker et al., 1992; Pasternack et al., 1997; Lizardi et al., 1998; Notomi et al., 2000; Deiman et al., 2002; Vincent et al., 2004). Among these methods, TMA, NASBA, RCA, and SDA cannot be considered truly isothermal because they require an initial heating step to denature the target nucleic acid before its amplification. Because no denaturation step is necessary to start amplification, RPA, HDA, and LAMP can be regarded genuinely isothermal. However, LAMP typically requires a reaction temperature of 60–65°C and three primer pairs, which may lead to primer-primer interactions that can limit the reaction. The main advantage of RPA over HAD been its speed, because it can amplify a single copy of nucleic acid to detectable levels in as little as 5–10 min. Furthermore, the use of both primers and a probe in the RPA reaction increases the specificity of the assay. We eliminated primer-dependent artifacts and avoided the formation of false-positive signals by introducing specific base substitutions into the primer and probe sequences and by rigorously screening and analyzing the formation of primer–probe complexes (Wu et al., 2020). The combination of RPA with the lateral flow immunoassay technique had the advantages of ease of detection, portability, and results that were readable with the naked eye. These advantages make RPA-LFS a method with which nucleic acids can be detected immediately.

Among the molecular targets utilized to identify *S. pneumoniae* were the Spn9802 fragment, the *recA* gene, the 16S rRNA gene, and virulence factor genes such as lysozyme (*ply*). Although these targets have shown beneficial in detecting *S. pneumoniae*, their capacity to identify it clearly remains a challenge. For example, both *ply* and Spn9802 have been associated with false-negative results (Abdeldaim et al., 2008; Carvalho Mda et al., 2007; El Aila et al., 2010; Zbinden et al., 2011). The autolysin gene *lytA* is highly conserved across *S. pneumoniae* strains, with only minor genetic change (0.11 percent–0.32 percent), and is found in practically all clinical isolates. As a result, it was chosen for the identification of *S. pneumoniae* in this case.

This RPA assay was highly specific and all 22 clinical isolates tested positive, whereas all 25 other common pathogens tested negative, indicating that the RPA-LFS established here specifically detected *S. pneumoniae*. A probit regression analysis was used to calculate the LOD (95% confidence level) of the method, which was 3.32 CFU per reaction. This is similar to the LOD of other highly sensitive molecular detection methods (Clancy et al., 2015; Wang et al., 2019).

In conclusion, we developed a sensitive and specific RPA-LFS assay for detecting *S. pneumoniae* in clinical specimens. Using the *lytA* gene as the diagnostic target, specific sets of primer-probe combinations were designed and screened. The detection of *S. pneumoniae* was completed within 30 min at 37°C. This assay had good potential utility for the detection of *S. pneumoniae* in resource-limited areas.

## Data Availability Statement

The original contributions presented in the study are included in the article/supplementary material. Further inquiries can be directed to the corresponding authors.

## Ethics Statement

The Medical Ethics Committee of Lianyungang City’s Second People’s Hospital examined and authorized the human-participant studies. To participate in the study, the patients/participants gave their written informed consent.

## Author Contributions

XG and GH designed the research. FW, YW, and XL conducted the research. CX, LW, and KW analyzed the data. The manuscript was written by FW and XG. The article was read and approved by all writers.

## Funding

This work was supported by the Jiangsu Province of China’s High-level Innovation and Entrepreneurship Talents Introduction Program (grant number 2019-30345), Lianyungang City’s ‘521 Project’ scientific research funding project (grant number LYG06521202157), Lianyungang City’s ‘HaiYan Plan’ scientific research funding project (grant number 2019-QD-008), and Jiangsu University’s Clinical Medical Science and Technology Development Fund (grant number JLY20180020).

## Conflict of Interest

The authors declare that the research was conducted in the absence of any commercial or financial relationships that could be construed as a potential conflict of interest.

## Publisher’s Note

All claims expressed in this article are solely those of the authors and do not necessarily represent those of their affiliated organizations, or those of the publisher, the editors and the reviewers. Any product that may be evaluated in this article, or claim that may be made by its manufacturer, is not guaranteed or endorsed by the publisher.
